# Epidemiological changes in clavicle fractures during the COVID-19 pandemic: a six-year analysis from a large single-center cohort

**DOI:** 10.1186/s13018-025-06634-x

**Published:** 2026-02-14

**Authors:** Nezih Ziroglu, Mehmet Utku Ciftci, Altug Duramaz, Cemal Kural, Ali Can Koluman

**Affiliations:** 1 Vocational School of Health Services, Department of Orthopedic Prosthetics and Orthotics, Acibadem Mehmet Ali Aydinlar University, 34638 Istanbul, Turkey; 2 Department of Orthopaedics and Traumatology, Acibadem University Atakent Hospital, 34307 Istanbul, Turkey; 3 Department of Orthopaedics and Traumatology, Abdulhamid Han Training and Research Hospital, Sultan 2, 34668 Istanbul, Turkey; 4 Department of Orthopaedics and Traumatology, Bakirkoy Dr. Sadi Konuk Training and Research Hospital, 34147 Istanbul, Turkey

**Keywords:** Clavicle fracture, Epidemiology, Upper extremity, Operative treatment, Conservative treatment, Collarbone, Lockdown, Pandemic, COVID-19

## Abstract

**Objective:**

To evaluate the epidemiological characteristics of clavicle fractures in a high-volume trauma center over a six-year period and to compare trauma mechanisms and management patterns before and during the COVID-19 pandemic.

**Methods:**

A retrospective review of 1500 consecutive clavicle fracture cases treated at a level-one trauma center between January 2016 and December 2021 was conducted. Demographic characteristics, fracture classification, trauma mechanisms, and treatment modalities were analyzed. Comparisons were made between the pre-pandemic period (2016–March 2020) and the pandemic period (March 2020–2021).

**Results:**

The cohort consisted predominantly of young males (68.5%, median age: 21.7 years). Midshaft fractures were most common (79.3%). During the pandemic, trauma mechanisms changed significantly, with a marked decrease in sports-related injuries (34.3% to 13.4%) and an increase in simple falls (46.0% to 62.9%) and high-energy trauma (19.7% to 23.7%) (*p* < 0.001). Despite these epidemiological shifts, the proportion of surgically treated fractures remained unchanged (8.7% pre-pandemic vs. 8.1% during the pandemic, *p* > 0.05). No significant differences were observed in fracture type or associated injuries between periods.

**Conclusion:**

The COVID-19 pandemic was associated with substantial changes in the epidemiology of clavicle fractures, particularly in trauma mechanisms, but did not affect the rate of operative versus non-operative management. These findings suggest that while societal restrictions altered injury patterns, clinical decision-making for clavicle fracture treatment remained stable.

**Level of evidence:**

III (Retrospective comparative study).

**Supplementary Information:**

The online version contains supplementary material available at 10.1186/s13018-025-06634-x.

## Introduction

Clavicle fractures constitute 2.6–5% of all fractures and represent one of the most frequent injuries of the shoulder girdle [1, 2]. Epidemiological studies from Western populations describe a bimodal age distribution in males—affecting both young adults and the elderly—whereas females typically demonstrate a unimodal peak in older age groups due to low-energy mechanisms [3, 4]. Most clavicle fractures involve the midshaft region, while distal and proximal fractures are less common [3, 5]. Trauma mechanisms vary with age, with simple falls predominating in older adults and sports- or traffic-related injuries being more common among younger individuals [6, 7].

Although the global epidemiology of clavicle fractures is well documented, data from Türkiye remain limited, and no large-scale study has examined epidemiological trends across the pre-pandemic and COVID-19 periods. The pandemic introduced profound changes in public behavior, mobility restrictions, and healthcare utilization, all of which have been shown to influence trauma patterns in various anatomical regions [8, 9]. Several reports demonstrated reductions in orthopedic emergency visits and alterations in injury mechanisms; however, findings regarding treatment practices have been heterogeneous among different trauma centers [6, 10, 21].

There remains a need for large, population-representative analyses evaluating how the pandemic influenced both the epidemiology and management of clavicle fractures. Türkiye, with its large urban population and high trauma burden, provides an important setting for such an investigation. Therefore, the objectives of this study were: (1) to analyze the etiological and epidemiological characteristics of 1500 clavicle fractures treated at a level-one trauma center over six years; and (2) to compare trauma mechanisms, fracture patterns, and management approaches between the pre-pandemic and pandemic periods.

We hypothesized that the COVID-19 lockdowns would be associated with significant changes in trauma mechanisms and seasonal distribution, while the proportion of operative versus non-operative treatment would remain stable.

## Methods

### Study design and setting

This retrospective cohort study was conducted at a high-volume, level-one trauma center in Istanbul, Türkiye. The institution is among the busiest orthopedic centers in the country, with over 2.3 million annual hospital visits and approximately 100,000 orthopedic consultations [11].

### Patient selection

All **consecutive** patients diagnosed with clavicle fractures between January 2016 and December 2021 were identified using the hospital’s digital archive. Inclusion criteria were:

1. Radiologically confirmed clavicle fracture, and.
2. Availability of complete clinical and demographic data.

Patients with missing demographic information or unavailable imaging were excluded. Of the 1758 screened patients, 1500 consecutive cases met the inclusion criteria and were analyzed.

Because the study involved all eligible cases within the defined period, a priori sample size or power calculation was not performed.

### Pandemic period definition

The first confirmed case of COVID-19 in Türkiye was reported on March 11, 2020, followed by nationwide lockdown measures beginning in April 2020 [12]. For comparison, cases were stratified into:

- **Pre-pandemic period**: January 2016 – March 10, 2020.
- **Pandemic period**: March 11, 2020 – December 2021.

### Variables and classification

The following variables were collected: age, sex, date of injury, trauma mechanism, laterality, season, associated injuries, fracture classification, and treatment method.

Trauma mechanisms were categorized as:

- **Simple falls (SF)**: Low-energy, same-level or domestic falls.
- **High-energy injuries (HI)**: Motor vehicle collisions, falls from height, motorcycle accidents.
- **Sports injuries (SI)**: All organized and recreational sports-related injuries.

Fractures were classified according to Robinson’s system (medial, midshaft, lateral) [2]. Treatment was dichotomized as **operative** (including delayed surgeries) or **non-operative**.

### Ethical considerations

The study received approval from the Clinical Research Ethics Committee of Bakırköy Sadi Konuk Training and Research Hospital, Istanbul (Approval No: 2019-14-02; Date: July 22, 2019). All procedures adhered to the Declaration of Helsinki.

### Statistical analysis

Statistical analyses were performed using NCSS (Number Cruncher Statistical System; Utah, USA). Continuous variables were presented as mean ¬± standard deviation or median (range) depending on distribution. Categorical variables were expressed as frequencies and percentages. Normality was assessed using the Shapiro-Wilk test and visual inspection of box plots. Comparisons between pre-pandemic and pandemic periods were performed using: Student’s t-test for normally distributed variables, Mann-Whitney U test for non-normal variables, Chi-square test, Fisher’s exact test, or Fisher-Freeman-Halton test for categorical variables. All statistical tests were two-tailed, and a* p*-value < 0.05 was considered statistically significant.* P*-values were reported for all comparative analyses, regardless of statistical significance, in accordance with good reporting practice.

## Results

### Demographics

A total of 1500 patients were included, of whom 1028 (68.5%) were male and 472 (31.5%) female (Table 1). The median age was 21.7 years (range: 0–92). Annual case counts gradually increased from 2016 to 2019, decreased in 2020, and rose again in 2021.

**Table 1:** Baseline demographic and injury characteristics of the study population

Characteristic		*N* or median	% or min-max
Sex	Female	472	31.5%
	Male	1028	68.5%
Age	Year	21.7	0–92
Year of trauma	2016	184	12.3%
	2017	208	13.9%
	2018	193	12.9%
	2019	308	20.5%
	2020	232	15.5%
	2021	375	25.0%
	Total pandemic	607	40.5%
Day of trauma	Monday	220	14.7%
	Tuesday	184	12.3%
	Wednesday	168	11.2%
	Thursday	199	13.3%
	Friday	201	13.4%
	Saturday	232	15.5%
	Sunday	296	19.7%
Weekday/Weekend	Weekday	999	66.6%
	Weekend	501	33.4%
Season of trauma	Spring	493	32.9%
	Summer	409	27.3%
	Autumn	320	21.3%
	Winter	278	18.5%
Affected side	Right	716	47.7%
	Left	784	52.3%
Radiological classification	Proximal	37	2.5%
	Shaft	1189	79.3%
	Distal	274	18.3%
Trauma mechanism	Simple fall	785	52.3%
	High-energy fall	320	21.3%
	Sports Injury	395	26.3%
Type of concomitant injury	Thorax injury	47	3.1%
	Abdominal injury	5	0.3%
	Ipsilateral injury	38	2.5%
	Contralateral injury	15	1.0%
	Vertebral column injury	11	0.7%
	Head trauma	15	1.0%
	Multitrauma	57	3.8%
	No trauma	1312	87.5%
Additional injury	Present	190	12.7%
	Absent	1310	87.3%
Treatment strategy	Surgical	127	8.5%
	Conservative	1373	91.5%
Time until surgery	Day	4	0–72
Reason for delayed surgery	Loss of reduction	12	9.4%
	Preop waiting time	94	74.0%
	Patient stabilization	21	16.5%

### Fracture characteristics

Left-side fractures were slightly more common (52.3%). Midshaft fractures comprised the majority (79.3%), followed by distal (18.3%) and proximal fractures (2.5%).

### Injury mechanisms and associated trauma

Simple falls were the most frequent mechanism (52.3%), followed by sports injuries (26.3%) and high-energy trauma (21.3%). Most fractures were isolated injuries (87.5%). Among patients with additional injuries, thoracic trauma (25%), ipsilateral extremity injuries (20%), and polytrauma (30%) were observed.

### Sex- and age-related patterns

Females sustained more simple-fall injuries (63.3%), whereas males sustained more high-energy trauma (25.2%) (*p* = 0.001).

Age differed significantly according to mechanism (*p* < 0.001):

- High-energy: 35.7 ± 17.1 years.
- Simple falls: 19.9 ± 24.3 years.
- Sports injuries: 12.8 ± 12.0 years.

Fracture type also varied by age (*p* < 0.001):

- Midshaft: 18.1 ± 19.5 years.
- Proximal: 37.2 ± 27.0 years.
- Distal: 36.3 ± 24.6 years.

Age groups demonstrated marked differences in trauma mechanism (*p* < 0.001).

Children/adolescents (≤ 18 years) sustained a high proportion of sports injuries (35.4%), whereas young adults (19–40 years) showed the highest rate of high-energy trauma (51.0%). Older adults (> 65 years) predominantly experienced simple falls (71.1%). (Supplementary Table 1)

### Mechanism changes during the COVID-19 pandemic

Of all fractures, 893 (59.5%) occurred before the pandemic and 607 (40.5%) during the pandemic. Age and sex distributions did not differ significantly between the two periods (*p* > 0.05).

However, trauma mechanisms changed significantly (*p* < 0.001):

- Sports injuries decreased (34.3% → 13.4%).
- Simple falls increased (46.0% → 62.9%).
- High-energy trauma increased slightly (19.7% → 23.7%).

Fracture types and associated injuries showed no significant differences between periods (*p* > 0.05). Seasonal distribution showed a modest shift with fewer spring presentations and relatively more summer/autumn cases during the pandemic (*p* = 0.001).

Although the proportion of high-energy injuries was slightly higher during the pandemic, the multivariable model adjusting for age and sex showed that the pandemic period was not an independent predictor of high-energy trauma (adjusted OR 1.29, 95% CI 0.99–1.69; *p* = 0.06).

### Treatment approaches

Overall, 127 patients (8.5%) underwent surgical fixation and 1373 (91.5%) were treated non-operatively. The rate of operative treatment did not differ significantly between the pre-pandemic and pandemic periods (8.7% vs. 8.1%, *p* > 0.05). Median time to surgery was 4 days (range: 0–72), with most delays related to preoperative logistics.

**Fig. 1:**
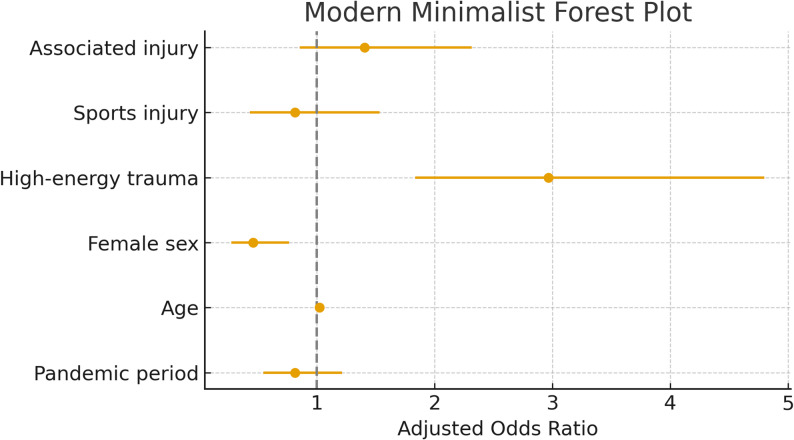
Modern minimalist forest plot demonstrating adjusted odds ratios (OR) and 95% confidence intervals for predictors of operative treatment. The pandemic period was not independently associated with increased likelihood of surgery after adjustment. Dashed vertical line indicates OR = 1

In the multivariable logistic regression adjusting for age, sex, trauma mechanism, and associated injuries, the pandemic period was not an independent predictor of operative treatment (adjusted OR 0.82, 95% CI 0.55–1.21; *p* = 0.31). Older age and high-energy trauma significantly increased the odds of surgery (*p* < 0.001), whereas female sex was associated with lower likelihood of operative treatment (*p* = 0.003). These findings confirm that clinical thresholds for recommending surgery remained unchanged during the pandemic (Table 2). These adjusted odds ratios are illustrated in Fig. 1.

**Table 2:** Multivariable predictors of surgical treatment

Variable	OR	CI_low	CI_high	*p*
Intercept	0.044	0.027	0.070	< 0.001
High-energy trauma	2.968	1.835	4.799	< 0.001
Sports injury	0.814	0.432	1.534	0.524
Pandemic	0.815	0.547	1.214	0.315
Age	1.022	1.013	1.031	< 0.001
Female	0.459	0.275	0.765	0.003
Associated injury	1.407	0.855	2.315	0.179

High-energy mechanism was the strongest predictor of associated injuries (adjusted OR 23.39, 95% CI 14.47–37.80; *p* < 0.001), while age also contributed (*p* < 0.001). The pandemic period did not increase the odds of presenting with associated trauma after adjustment (*p* = 0.64). Among the 127 surgically treated patients, 27 (21%) underwent surgery more than 7 days after injury. In the adjusted analysis, the pandemic period was not associated with increased odds of delayed surgery (adjusted OR 0.55, 95% CI 0.20–1.56; *p* = 0.26). Older age was the only significant predictor of delay (*p* = 0.001).

## Discussion

This study presents one of the largest single-center series of clavicle fractures reported from Türkiye and provides a detailed comparison of epidemiological characteristics before and during the COVID-19 pandemic. The most notable finding was a significant shift in trauma mechanisms during the pandemic period, whereas the proportion of operative versus non-operative treatment remained unchanged. These results indicate that while societal and behavioral changes associated with lockdowns altered the context in which injuries occurred, core clinical decision-making for clavicle fracture management was stable.

The demographic distribution in our cohort—male predominance and a high incidence in young adults—is consistent with prior epidemiological reports [1, 5]. Similarly, the predominance of midshaft fractures aligns with previous studies [2, 4]. Prior literature has shown that simple falls tend to occur in older adults, whereas sports and traffic-related injuries are more common in younger populations [3, 5]. In our study, sports-related injuries decreased markedly during the pandemic, paralleling the widespread closure of sports facilities and restrictions on organized physical activity reported elsewhere [6, 9].

A major strength of the present dataset is the detailed evaluation of trauma mechanism across age groups. Our age-stratified analysis demonstrated distinct injury patterns, with sports injuries representing more than one-third of fractures in patients ≤ 18 years, high-energy trauma peaking in young adults (19–40 years), and simple falls dominating in older adults (> 65 years). This pattern mirrors international epidemiological trends and underscores the need for age-adapted injury prevention strategies, particularly during periods of societal disruption [5, 18].

High-energy trauma showed a modest increase during the pandemic, which may be related to greater reliance on delivery services and the corresponding rise in motorcycle traffic reported in other regional and global analyses. However, multivariable analysis demonstrated that the pandemic period was **not** an independent predictor of high-energy trauma after adjusting for age and sex. Furthermore, neither fracture type nor the presence of associated injuries differed significantly between periods. Logistic regression confirmed that high-energy mechanism—not the pandemic period—was the dominant predictor of associated injuries (OR > 20), suggesting that the mechanism shift altered the distribution of injury contexts rather than the overall severity profile [14, 15].

A key finding of this study is that the pandemic did not influence surgical decision-making. Although unadjusted surgical rates were similar between periods (8.7% vs. 8.1%), multivariable logistic regression adjusting for age, sex, mechanism, and associated injuries confirmed that the pandemic period was *not* an independent predictor of operative management. Older age and high-energy trauma increased the odds of surgery, whereas female sex reduced the likelihood of operative intervention. These results indicate that despite shifts in injury mechanisms, established criteria for operative fixation were consistently applied [19]. This aligns with reports from centers that maintained routine trauma services during COVID-19 [9, 6] and contrasts with studies showing reduced surgical volume due to resource reallocation [2, 13, 16, 17, 22].

Delayed surgery was another relevant consideration during the pandemic. In our cohort, 21% of surgically treated patients underwent fixation more than 7 days after injury. However, after adjustment, the pandemic period was not associated with increased odds of delayed surgery; age was the only significant predictor of delay. This suggests that institutional capacity and workflow adaptations were sufficient to prevent treatment backlog despite fluctuating trauma volumes.

The present study contributes valuable regional data, as epidemiological analyses of adult clavicle fractures from Türkiye remain limited. Previous national reports have focused primarily on pediatric trauma [18]. However, as King et al. demonstrated, fracture patterns may differ between developing and developed countries due to variations in trauma exposure and healthcare access [16, 23]. Türkiye, as an upper-middle-income country with high urban mobility, represents a unique context; our data support the notion that even well-resourced centers can experience significant shifts in trauma mechanism during major societal disruptions.

Although operative fixation for displaced midshaft clavicle fractures has been increasingly adopted worldwide [4, 20], the pandemic presented a natural experiment to evaluate whether external constraints altered clinical thresholds. In our institution, surgical proportions remained stable—even after multivariable adjustment—indicating that standard indications were preserved through maintained staffing, workflow continuity, and consistent senior oversight. This is consistent with recent systematic reviews emphasizing that treatment should continue to be individualized based on fracture pattern, displacement, and patient-specific factors [1, 10].

This study has important strengths, including its large sample size, radiological confirmation of all cases, and consistent institutional protocols over a six-year period. However, several limitations must be acknowledged. The retrospective design introduces the possibility of reporting bias. Functional outcomes, complications, and quality-of-life measures were not assessed, limiting conclusions regarding the long-term comparative effectiveness of treatment strategies. Additionally, as a single-center study, the results may not fully represent national epidemiological patterns, although the high trauma volume of our institution likely reflects urban Türkiye.

## Conclusion

This large single-center study demonstrates that the COVID-19 pandemic led to notable shifts in the epidemiology of clavicle fractures, particularly through a marked reduction in sports-related injuries and an increase in both simple falls and high-energy trauma. Despite these changes in injury patterns, the overall proportion of surgically treated fractures remained stable, and the pandemic period was not an independent predictor of operative management in multivariable analysis. These findings indicate that clinical decision-making for clavicle fracture treatment—guided by established indications—was preserved even under the altered societal conditions of the pandemic. Future multicenter and prospective investigations are warranted to determine whether these epidemiological shifts have lasting effects on clinical practice, patient outcomes, and healthcare resource allocation.

## Supplementary Information

Supplementary Material 1.

## Data Availability

The datasets generated and analyzed during the current study are available from the corresponding author upon reasonable request.
