# Supplementary material for: Epidemiological changes in clavicle fractures during the COVID-19 pandemic: a six-year analysis from a large single-center cohort
Source: J Orthop Surg Res. 2026 Feb 14;21:209. doi: 10.1186/s13018-025-06634-x (PMC13011530; doi:10.1186/s13018-025-06634-x)
Supplement: Supplementary file 1 — Supplementary Material 1. [file 13018_2025_6634_MOESM1_ESM.docx]

# Supplementary Table 1. Trauma Mechanism by Age Group

| Age Group | Simple fall (1) | High-energy (2) | Sports injury (3) |
| --- | --- | --- | --- |
| ≤18 | 529 | 50 | 317 |
| 19–40 | 85 | 150 | 59 |
| 41–65 | 115 | 103 | 6 |
| >65 | 54 | 17 | 5 |
